# Improving Patient Care and Clinical Services: Compliance With the British Orthopaedic Association Standards for Trauma (BOAST) Guidelines for Neurological and Vascular Assessment of Acute Fractures

**DOI:** 10.7759/cureus.98439

**Published:** 2025-12-04

**Authors:** Raja Muhammad Mussab, Irfan Ahmad, Rida Sohail, Tassreena Jahan Jany, Vishnu Raja Anil Kumar

**Affiliations:** 1 Orthopaedics and Trauma, Jinnah Postgraduate Medical Centre, Karachi, PAK; 2 Orthopaedics and Trauma, Russell Hall Hospital, Dudley, GBR

**Keywords:** clincal audit, clinical examination, neurological examination, quality improvement and patient safety, vascular examination

## Abstract

Introduction

Traumatic limb fractures risk neurovascular (NV) compromise and require documented nerve-specific sensory, motor, and arterial assessments to support early detection and management. The British Orthopaedic Association Standards for Trauma (BOAST) mandate clear documentation of peripheral nerve function after injury, manipulation, cast fitting or surgery. This closed-loop audit measured current practice against the December 2021 BOAST guideline and tested whether a targeted educational intervention could improve NV documentation.

Methods

An audit for service evaluation and quality improvement at Russell Hall Hospital, UK, reviewed 97 adults admitted with acute upper- or lower-limb fractures across two cycles (49 patients, January 2024; 48 patients, July 2024). A single educational awareness presentation took place in May 2024. Data were extracted from Trauma & Orthopaedic (T&O) admission notes and benchmarked against BOAST criteria requiring specific nerve and arterial status. Median age and interquartile range (IQR) were reported; categorical comparisons used Pearson’s χ², and effect size was reported as phi (φ). Significance threshold was p < 0.05.

Results

Median age was 64 years (IQR: 34.5-79.5). The use of NV documentation rose from 87.8% to 97.9% following the intervention (χ² = 3.74, df = 1; p = 0.053). Detailed neurological recording increased from 38.8% to 54.2% (χ² = 2.31, p = 0.129; φ = 0.154). Detailed vascular recording improved from 26.5% to 45.8%, reaching statistical significance (χ² = 3.92, p = 0.048; φ = 0.201). Many entries continued to use the nonspecific phrase 'NV intact' rather than specific details.

Conclusion

Educational sessions within a closed-loop audit framework improved completion of an NV assessment and a significant rise in vascular details, while neurological details showed a slight increase. Continued adherence to BOAST guidance and structured feedback mechanisms should preserve high standards of NV assessment and improve patient safety.

## Introduction

Traumatic musculoskeletal injuries of the upper and lower limbs are common in acute hospital settings, yet their consequences extend beyond bony fractures to include potential neurovascular (NV) compromise [1]. A comprehensive neurological and vascular assessment of any injured limb is a central component of initial assessment and subsequent care. Thorough documentation of nerve-specific sensory and motor function and arterial status is essential to detect early neuropathic or vascular injury [2]. British Orthopaedic Association Standards for Trauma (BOAST) requires that a full assessment of all relevant peripheral nerve functions be performed and clearly documented as soon as possible after injury, manipulation, cast fitting, or surgery [3]. Despite this guidance, NV assessment documentation remains inconsistent in many services. A recent audit at a UK major trauma centre reported that only about 40% of ankle-fracture patients had NV documentation compliant with BOAST standards [4].

Failure to undertake or record a detailed NV examination at presentation, after reduction or following fixation may result in missed peripheral nerve injury, delayed referral, or inappropriate management pathways [5]. Mis- or underdocumented nerve injuries in orthopaedic trauma carry serious clinical consequences, including irreversible deficit, chronic pain, functional impairment, and limb amputation, with psychological burden for patients [6]. From a systems perspective, such oversights increase length of stay, prompt re-intervention, expand rehabilitation needs, and raise the risk of litigation, all of which impair service efficiency and governance.

Given these imperatives, the present study audited NV documentation for patients sustaining upper- and lower-limb fractures at a UK hospital and benchmarked practice against the December 2021 BOAST 'Peripheral Nerve Injury' guideline. Targeted corrective measures comprised educational awareness via a presentation about the consequences of poor NV assessment. The study used a closed-loop audit design in which measurement-informed interventions and subsequent re-audit were done to identify practice gaps and evaluate the effect of educational intervention.

## Materials and methods

This observational, closed-loop audit was conducted at Russell Hall Hospital, UK, and reviewed acute fracture admissions during two discrete cycles. Ethical approval was obtained by registration in the Trust's official Portal called Audit Management and Tracking (AMAT), and anonymised data were used to maintain confidentiality. 

The inclusion criteria comprised adult patients presenting to the Emergency Department with acute fractures of the upper or lower limb, with the subsequent orthopaedic admission clerking documented in the Trauma & Orthopaedic (T&O) admission records. Patients were excluded if they had chronic fractures, non-traumatic fractures (such as pathological fractures), or if they were admitted via ward referral rather than direct Emergency Department presentation. 

A total of 97 patients were included. The first cycle comprised 49 patients (24 upper‐limb, 25 lower‐limb) in January 2024; the second (re-audit) cycle comprised 48 patients (25 upper‐limb, 23 lower‐limb) in July 2024 following implementation of corrective interventions in May 2024. 

Data were extracted from the T&O admission documentation and assessed for the presence of detailed neurovascular (NV) examination and documentation. Each case was benchmarked against the standards set out in the British Orthopaedic Association’s BOAST guideline for peripheral nerve injury, with criteria requiring documentation of both specific nerve and arterial status for the affected region as per local protocol. The target was 100% compliance with the standard. 

Descriptive statistics were used to report age as median and interquartile range, and other categorical variables were presented as counts and percentages. Comparisons of proportions between the two audit cycles (NV documentation usage, neurological details recorded, vascular details recorded) were performed using Pearson’s chi-square test for 2 × 2 contingency tables. Effect size for 2 × 2 comparisons was reported as the phi coefficient (equivalent to Cramer’s V), with values interpreted to indicate the magnitude of association. All tests were two-sided, and a significance threshold of p < 0.05 was applied. Analyses were performed using IBM SPSS Statistics for Windows, Version 27 (Released 2019; IBM Corp., Armonk, New York, United States). 

## Results

A total of 97 patients were included in the audit, drawn from two discrete cycles, with 49 patients in the first cycle and 48 in the re-audit cycle. The median age was 64 years (IQR: 34.5-79.5 years). The cohort, therefore, represented a predominantly older adult population, although a wide spread of ages was present. Regarding injury, 53 patients (54.6%) had right-sided injuries, 43 patients (44.3%) had left-sided injuries, and one patient (1.0%) had bilateral fractures. Upper-limb fractures and lower-limb fractures occurred in almost equal numbers, with 49 patients (50.5%) sustaining upper-limb injuries and 48 patients (49.5%) sustaining lower-limb injuries (Table 1). These baseline demographics and injury distributions set the context for the subsequent analysis of NV documentation. 

**Table 1:** Demographic and injury characteristics of the study population (n = 97) IQR: interquartile range

Variable	Frequency (%) or median (IQR)
Age (years)	64 [34.5–79.5]
Injury side	
Right	53 (54.6%)
Left	43 (44.3%)
Bilateral	1 (1.0%)
Injury region	
Upper limb	49 (50.5%)
Lower limb	48 (49.5%)

Table 2 presents that the use of NV documentation improved from 87.8% in the first cycle to 97.9% in the second, demonstrating a marked improvement following the implemented interventions. However, it is important to note that the documentation mentioned 'NV intact' only. Therefore, it was not detailed enough on the NV findings. This change in using the NV documentation between cycles did not reach conventional statistical significance on the Pearson χ² test (χ² = 3.74, df = 1; p = 0.053). After corrective intervention, a substantial increase was observed in the recording of detailed neurological findings, which rose from 38.8% to 54.2%. This change did not reach statistical significance (Pearson χ² = 2.31, p = 0.129; φ = 0.154). The small effect size indicates a weak association between the cycle and the neurological recording. Similarly, documentation of detailed vascular assessment improved from 26.5% to 45.8%. Vascular documentation improved to a greater extent and reached the conventional threshold for significance on the Pearson χ² test (χ² = 3.92, p = 0.048; φ = 0.201). The effect size remains small, suggesting a modest association. The two audit cycles showed a clear directional improvement in both neurological and vascular documentation, but the strength of evidence was weak. These results suggest that the measure to enhance the awareness associated with NV examination between the audit cycles was marginally effective in increasing the completeness and quality of NV records in fracture admissions, which is in line with the BOAST standards. 

**Table 2:** Neurovascular documentation and statistical comparison between audit cycles (n = 97) χ²: Pearson chi-square; φ: phi coefficient (effect size); *significance level = <0.05

Outcome	Cycle	Yes (n, %)	No (n, %)	χ²	p-value	Effect size (φ)
NV documentation	1st cycle (Jan 2024)	43 (87.8%)	6 (12.2%)	3.74	0.053	0.196
	2nd cycle (Jul 2024)	47 (97.9%)	1 (2.1%)			
Neurological details recorded	1st cycle (Jan 2024)	19 (38.8%)	30 (61.2%)	2.31	0.129	0.154
	2nd cycle (Jul 2024)	26 (54.2%)	22 (45.8%)			
Vascular details recorded	1st cycle (Jan 2024)	13 (26.5%)	36 (73.5%)	3.92	0.048	0.201
	2nd cycle (Jul 2024)	22 (45.8%)	26 (54.2%)			

## Discussion

The audit showed a clear improvement in the overall use of NV documentation, rising from 87.8% to 97.9%. However, many entries still contained the brief phrase ‘NV intact’ instead of specific neurological or vascular details. Recording of detailed neurological findings increased modestly, while documentation of vascular assessment showed a more notable improvement. Although both areas demonstrated progress, the overall strength of change was limited, suggesting that while interventions encouraged better recording practices, further reinforcement and education are still needed to achieve consistent, detailed documentation. Similar to this, Gilmore noted that clinicians recorded NV status using the phrase ‘neurovascularly intact’, which provides no detailed information [7]. Our audit cycles reflected the same shortcoming, as NV documentation was often used but lacked the neurological and vascular detail necessary for clinical decision-making. 

These results compare favourably with the baseline reported in a UK major trauma-centre audit that found approximately 40% compliance with BOAST standards for ankle fractures [4]. The near-universal adoption of NV documentation in the re-audit suggests that awareness and workflow changes increased the likelihood of completing a record, yet completion did not always equate to clinical detail. The pattern of greater gains in vascular recording than in neurological detail is consistent with studies that show vascular elements are more frequently omitted in routine practice unless prompts or specific fields require their entry [8,9]. The significant improvement in vascular documentation here likely reflects the targeted educational content combined with the proforma prompt introduced between cycles. 

Educational interventions commonly aid retention of sensory and motor examination techniques, particularly among junior doctors and emergency staff, and that mechanism plausibly underpins the directional gains observed here [10,11]. Harper et al. and Mohamed et al. report similar closed-loop audit approaches that produced meaningful improvements when teaching was paired with standardised documentation tools [12,13]. Electronic templates and embedded reminders further reduce reliance on memory and support consistent practice. McKee et al. and other quality-improvement work describe improved completeness and legibility when proformas or templates are used [14,15]. The present audit’s electronic admission template likely contributed to the rise in documented vascular checks and the overall increase in NV documentation. 

Clinical workload and staffing pressures remain important contextual factors. Hashim et al. found lower compliance during busy trauma lists and when senior supervision was limited [16], a finding that matches the plausible drivers of remaining omissions in this audit. The small sample and short interval between cycles increase the risk of type II error for the neurological outcome; observed p = 0.129 may reflect inadequate power rather than absence of effect. Retrospective review means results depend on what was written rather than on observed practice, so improved documentation may not always indicate improved examination quality. The findings indicate that a focused educational intervention can raise the frequency of NV documentation and produce a statistically significant improvement in vascular recording, while gains in neurological detail remain directionally positive but did not reach significance in this sample. 

Limitations include a modest sample size, retrospective record review, and a short follow-up between cycles, which restricts generalisability and leaves uncertain whether improved documentation translates into better patient outcomes. Future work should involve multiple centres, larger cohorts, and measures linking documentation quality to clinical outcomes such as nerve recovery, limb function, or time to intervention. 

## Conclusions

This audit demonstrated clear improvement in NV documentation after educational intervention, with a significant rise in detailed vascular recording and a slight increase in neurological detail recording. Consistent effort to improve awareness among clinicians with the help of educational intervention, combined with an electronic proforma and routine trauma-meeting review, moves local practice closer to BOAST expectations. An expansion of the audit to multiple centres and linking documentation quality to patient outcomes would strengthen the evidence base and promote national consistency in trauma care. Continued adherence to BOAST guidance and structured feedback mechanisms should preserve high standards of NV assessment, improve patient safety, support better clinical decision-making, and enable prompt escalation when concerns arise. 
